# MDA5 and TLR3 Initiate Pro-Inflammatory Signaling Pathways Leading to Rhinovirus-Induced Airways Inflammation and Hyperresponsiveness

**DOI:** 10.1371/journal.ppat.1002070

**Published:** 2011-05-26

**Authors:** Qiong Wang, David J. Miller, Emily R. Bowman, Deepti R. Nagarkar, Dina Schneider, Ying Zhao, Marisa J. Linn, Adam M. Goldsmith, J. Kelley Bentley, Umadevi S. Sajjan, Marc B. Hershenson

**Affiliations:** 1 Department of Molecular and Integrative Physiology, University of Michigan Medical School, Ann Arbor, Michigan, United States of America; 2 Department of Internal Medicine, University of Michigan Medical School, Ann Arbor, Michigan, United States of America; 3 Department of Microbiology and Immunology, University of Michigan Medical School, Ann Arbor, Michigan, United States of America; 4 Department of Pediatrics and Communicable Diseases, University of Michigan Medical School, Ann Arbor, Michigan, United States of America; Washington University School of Medicine, United States of America

## Abstract

Rhinovirus (RV), a single-stranded RNA picornavirus, is the most frequent cause of asthma exacerbations. We previously demonstrated in human bronchial epithelial cells that melanoma differentiation-associated gene (MDA)-5 and the adaptor protein for Toll-like receptor (TLR)-3 are each required for maximal RV1B-induced interferon (IFN) responses. However, *in vivo*, the overall airway response to viral infection likely represents a coordinated response integrating both antiviral and pro-inflammatory pathways. We examined the airway responses of MDA5- and TLR3-deficient mice to infection with RV1B, a minor group virus which replicates in mouse lungs. MDA5 null mice showed a delayed type I IFN and attenuated type III IFN response to RV1B infection, leading to a transient increase in viral titer. TLR3 null mice showed normal IFN responses and unchanged viral titers. Further, RV-infected MDA5 and TLR3 null mice showed reduced lung inflammatory responses and reduced airways responsiveness. Finally, RV-infected MDA5 null mice with allergic airways disease showed lower viral titers despite deficient IFN responses, and allergic MDA5 and TLR3 null mice each showed decreased RV-induced airway inflammatory and contractile responses. These results suggest that, in the context of RV infection, binding of viral dsRNA to MDA5 and TLR3 initiates pro-inflammatory signaling pathways leading to airways inflammation and hyperresponsiveness.

## Author Summary

Rhinovirus (RV) is the most frequent cause of acute respiratory tract infection in humans. RV has emerged as the most frequent pathogen associated with exacerbations of asthma. However, the mechanisms by which RV causes asthma flare-ups are not precisely known. We studied the requirements of two receptors which bind viral double-stranded RNA, melanoma differentiation-associated gene (MDA)-5 and Toll-like receptor (TLR)-3, for RV-induced airway responses using specific knockout mice. We found that MDA5 and TLR3 deficiencies had modest effects on viral titer. However, MDA5 and TLR3 knockout mice showed significantly reduced airway inflammation and responsiveness in response to RV infection. Mice with allergic airways disease also showed reduced airway responses. These results suggest that, in the context of RV infection, TLR3 and MDA5 initiate pro-inflammatory signaling pathways which lead to airways inflammation and hyperresponsiveness.

## Introduction

Rhinovirus (RV) is the most frequent cause of acute respiratory tract infection in humans. RV infection is typically responsible for upper respiratory symptoms including rhinorrhea, sore throat, nasal congestion, sneezing, cough, and headache. More importantly, RV has emerged as the most frequent pathogen associated with exacerbations of asthma [1], [2].

RV is a positive sense single-stranded RNA (ssRNA) virus from the *Picornaviridae* family. After endocytosis, RV RNA is inserted into the cytosol, where viral replication occurs. During replication, a double-stranded RNA (dsRNA) intermediate is formed which represents an important stimulus of the host innate immune response. The mechanisms by which RV causes asthma exacerbations are not fully established, but current evidence indicates that the immune response is critical in this process. The first line of defense against RV infection is the innate immune system. Innate pathogen sensors detect viral products and respond by initiating a signaling cascade that leads to a rapid antiviral response involving secretion of interferons (IFNs) and inflammatory cytokines. RV induces the expression of both type I (*e.g.*, IFN-α, IFN-β) and type III IFNs (in mice, IFN-λ2/IL-28A, IFN-λ3/IL-28B). Though they signal through the engagement of different receptor complexes, the intracellular signaling program activated by type I and type III IFNs is very similar, as evidenced by the cellular gene expression profiles induced after stimulation with IFN-λ versus IFN-α [3]. However, whereas the type I receptor complex is expressed ubiquitously, the type III receptor is only expressed in few cell types, notably epithelial cells [4]. The preferential expression of IFN-λ receptors on epithelial surfaces may allow the host to rapidly control and perhaps eliminate viruses at the major portals of entry into the body before infection is established, and without activating other arms of the immune system.

Several pattern recognition receptors have been shown to be responsible for binding viral dsRNA and initiating the IFN response. Retinoic acid inducible gene (RIG)-I and melanoma differentiation-associated gene (MDA)-5 are homologous proteins located within the cytoplasm, whereas Toll-like receptor (TLR)-3 is located mainly on the endosomal membrane and plasma membrane. RIG-I has been shown to preferentially recognize 5′ phosphorylated short dsRNA, whereas MDA5 recognizes long dsRNAs [5], [6], [7], [8]. Thus, RIG-I has been shown to detect negative-strand viruses such as influenza, respiratory syncytial virus (RSV) and paramyxovirus [9], as well as some positive-strand flaviviruses. In comparison, MDA5 has been shown to selectively detect positive-strand viruses including picornaviruses (encephalomyocarditis virus, Mengo virus and Theiler's virus). Previously, we demonstrated in cultured human bronchial epithelial cells that MDA5 and TIR-domain-containing adapter inducing interferon-β (TRIF), the adaptor protein for TLR3, are each required for maximal RV1B-induced IFN responses [10]. Knockdown of RIG-I had no effect on IFN responses. TRIF, but not MDA5, was required for maximal pro-inflammatory cytokine expression [10]. TLR3 is partially required for HRV39-induced IL-8 expression in 16HBE14o- human bronchial epithelial cells [11], as well as HRV1A-induced MUC5AC expression in NCI-H292 mucoepidermoid carcinoma cells [12].

However, *in vitro* studies may not truly represent the complicated situation *in vivo*, where multiple cell types are involved. Further, the overall airway response to viral infection likely represents a coordinated response integrating both antiviral and pro-inflammatory pathways. For example, it has been proposed that asthmatics are susceptible to RV infection due to deficient IFN production. RV-infected airway epithelial cells from asthmatic subjects show impaired production of IFN-β and -λ [13], [14] and asthmatics experimentally infected with RV16 showed a reduced IFN-γ/IL-5 mRNA ratio in their sputum [15]. According to the theory, reduced IFN responses, in turn, lead to increased viral-mediated inflammation. However, it is also conceivable that reduced RV-induced IFN responses are coupled with attenuated airways inflammation and responsiveness. For example, pneumovirus-infected IFNα/β receptor null mice show fewer BAL leukocytes and prolonged survival despite increased virus titers [16]. To address these questions, we examined the airway responses of TLR3- and MDA5-deficient mice to infection with RV1B, a minor group virus which replicates in mouse lungs [17].

## Results

### Effects of MDA5 and TLR3 deficiencies on RV1B-induced type I and type III IFNresponses

**Figure 1:**
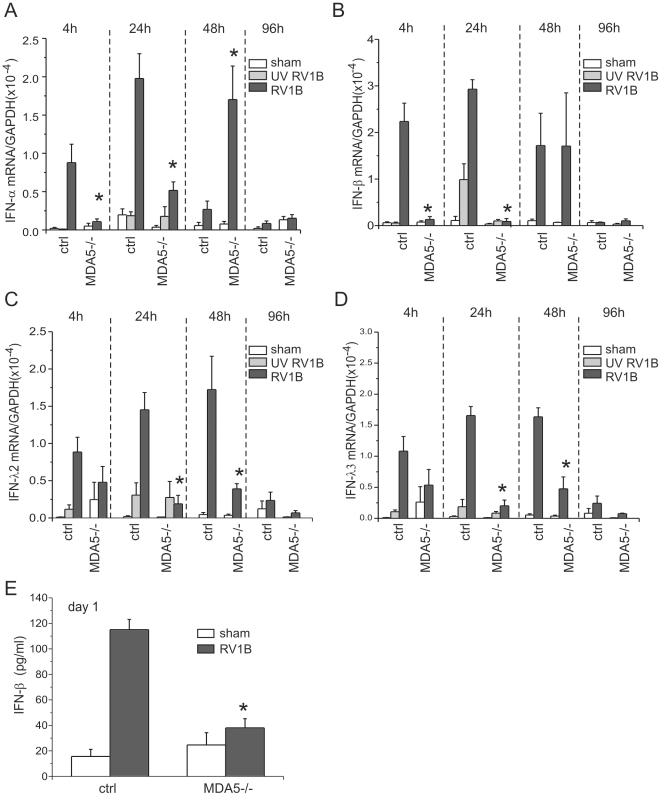
RV1B-induced expression of type I and III IFNs in MDA5−/− mice. MDA5−/− and their control mice were inoculated with sham, UV-irradiated RV1B (UV RV1B) or intact RV1B. Lungs were harvested at 4, 24, 48, and 96 h after infection. A–D. The expression of IFN-α, IFN-β, IFN-λ2 and IFN-λ3 at each time point was determined by qPCR. E. IFN-β protein production was measured by ELISA at 24 h post-infection. The expression of each target gene was normalized to GAPDH. Data represent mean±SEM for 3–7 mice.

Our previous studies in cultured airway epithelial cells demonstrated that MDA5 and TRIF, the adaptor protein for TLR3, are required for maximal RV-induced IFN expression. However, while the airway epithelium is a major target of RV infection, other cell types such as monocytes and macrophages may also be infected [18]. To determine whether MDA5 and TLR3 are required for RV1B-induced airway IFN responses *in vivo*, we studied MDA5−/− mice, TLR3−/− mice, and their strain-matched controls. Mice were inoculated intranasally with sham HeLa cell lysate, replication-deficient UV-irradiated RV1B or intact RV1B. Lungs were harvested at 4–96 h after infection. RV1B infection caused a significant induction of the expression of type I IFNs (IFN-α and IFN-β) in control mice compared to sham or UV-RV1B inoculated mice. However, MDA5−/− mice showed minimal type I IFN responses 4 and 24 h post-infection (Figures 1a, 1b). The IFN-β protein response 24 h after infection was also significantly decreased in MDA5−/− mice compared to control mice (Figure 1e). Interestingly, by 48 h post infection, when the type I IFN expression in RV1B-infected control mice was essentially over, MDA5−/− mice showed robust but delayed type I IFN response. In contrast, the expression of type III IFNs (IFN-λ2 and IFN-λ3) in RV1B-infected MDA5−/− mice was significantly lower than that of control mice throughout the time course studied (Figures 1c, 1d). These data suggest that the regulation of type I and type III IFN differ in the context of RV infection.

In contrast to MDA5 null mice, there was no difference in type I IFNs or type III IFNs expression between TLR3−/− mice and controls (Figures S1a–e). IFNs-α, -β, -λ2 and -λ3 each showed brisk responses following RV1B infection of TLR3−/− mice. Taken together, these data suggest that both type I and type III IFNs are partially dependent on MDA5, but not TLR3, for their induction.

### MDA5 regulates viral titer

**Figure 2:**
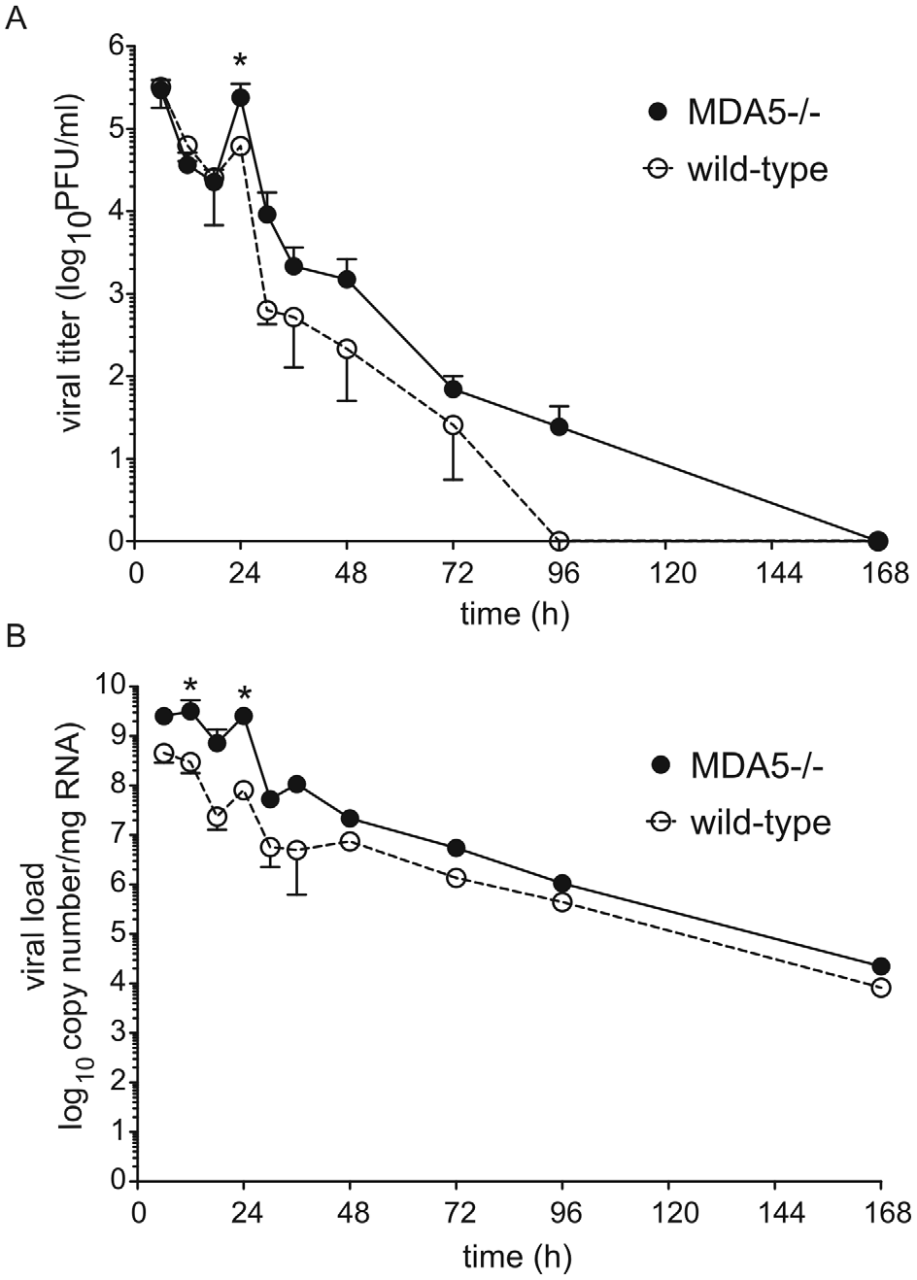
RV1B titer and copy number in MDA5−/− mice. MDA5−/− and their control mice were infected with RV1B. Lungs were harvested at 4, 24, 48, and 96 h after infection. A. Lung titer at 24 h post-infection was determined by plaque assay. B. RV1B copy number at each time point was determined by qPCR. RV copy number was normalized to 18S rRNA. Data represent mean±SEM for 3–7 mice (*p<0.05, one-wayANOVA).

To determine whether MDA5 plays a role in viral clearance, we determined lung viral titer and copy number by plaque assay and qPCR, respectively. As we have observed previously, control mice showed a modest increase in lung viral titer and copy number 24 h after infection [17], indicating a small amount of viral replication. There was also a small but statistically significant increase in viral titer and copy number in MDA5−/− mice compared to that of control mice 24 h post infection (Figures 2a, 2b). Viral titer and copy number were not significantly different between the two groups at 48 h. Thus, MDA5−/− mice demonstrate a transient increase in infectious virus, perhaps due to the delayed type I and reduced type III IFN response.

There was no difference in viral titer or copy number between the control and TLR3−/− mice (Figures S2a, S2b). The absence of a change in viral load is consistent with the normal IFN response observed in TLR3−/− mice.

### MDA5 and TLR3 signaling are each required for RV1B-induced inflammatory responses

**Figure 3:**
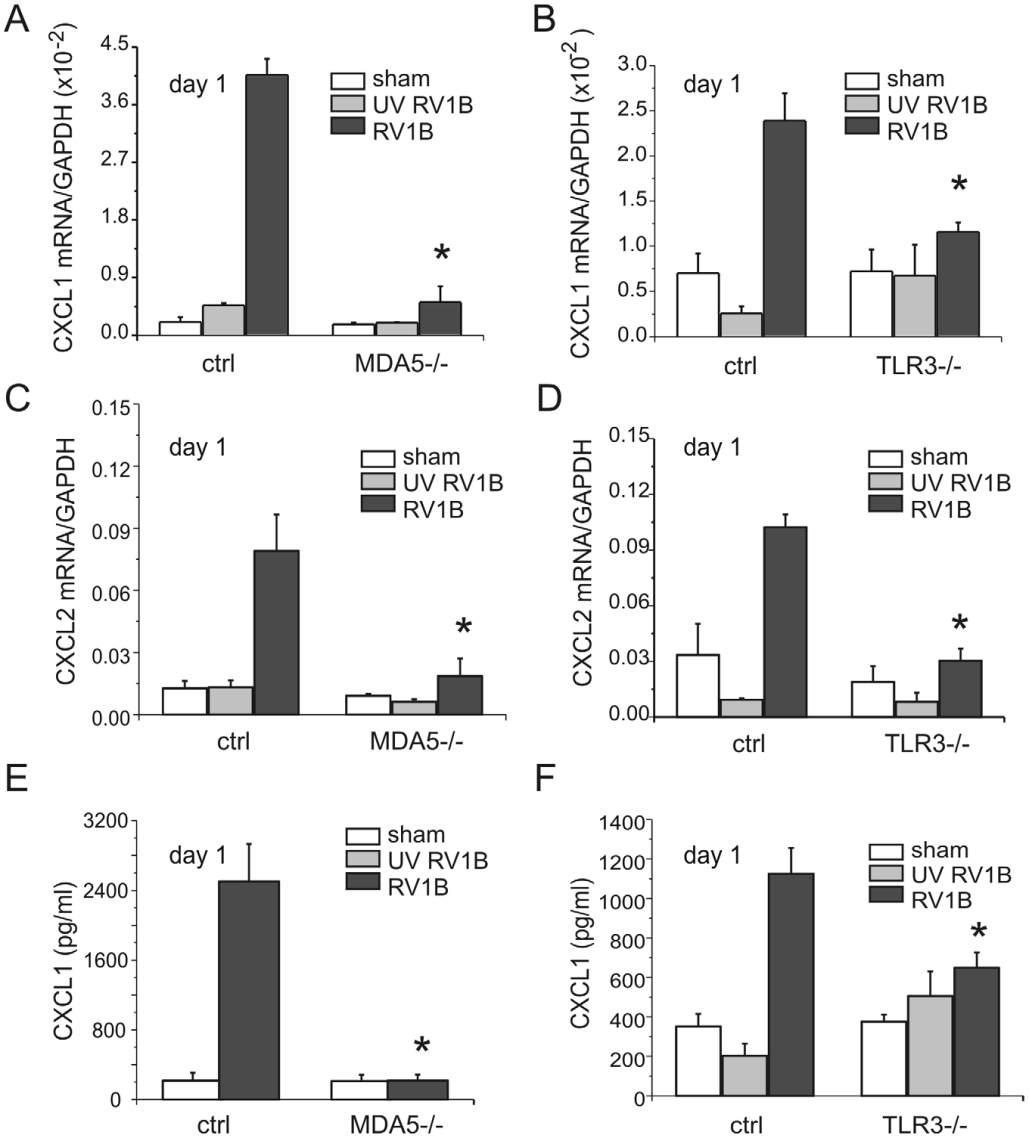
RV1B-induced chemokine expression in MDA5−/− and TLR3−/− mice. MDA5−/−, TLR3−/− and their strain control mice were inoculated with sham, UV-irradiated RV1B (UV RV1B) or intact RV1B. Lungs were harvested 24 h after infection. A–D. The expression of CXCL1/KC, CXCL2/MIP-2, CCL2/MCP-1, CXCL10/IP-10 and CCL11/eotaxin-1 was determined by qPCR. E–F. Protein production of CXCL1/KC and CXCL2/MIP-2 was measured by ELISA and bioplex assay. The expression of each target gene was normalized to GAPDH. Data represent mean±SEM for 3–7 mice (*p<0.05, one-way ANOVA).

**Figure 4:**
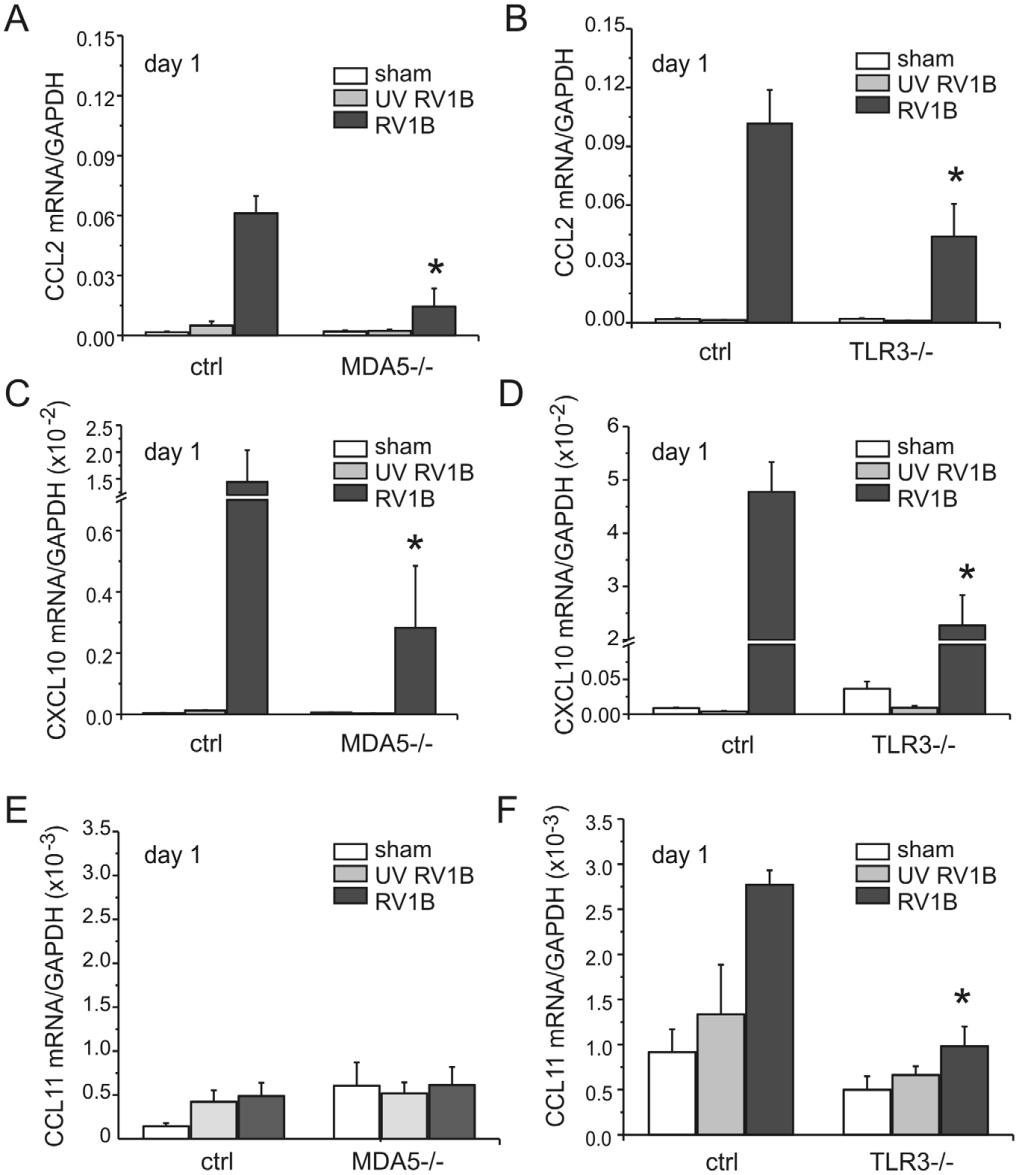
RV1B-induced pro-inflammatory cytokine expression in MDA5−/− and TLR3−/− mice. MDA5−/−, TLR3−/−, and their strain control mice were inoculated with sham, UV-irradiated RV1B (UV RV1B) or intact RV1B. Lungs were harvested 24 h after infection. A–F. The expression of CXCL1/KC, CXCL2/MIP-2, CCL2/MCP-1, CXCL10/IP-10 and CCL11/eotaxin-1 was determined by qPCR. The expression of each target gene was normalized to GAPDH. Data represent mean±SEM for 3–7 mice (*p<0.05, one-way ANOVA).

**Figure 5:**
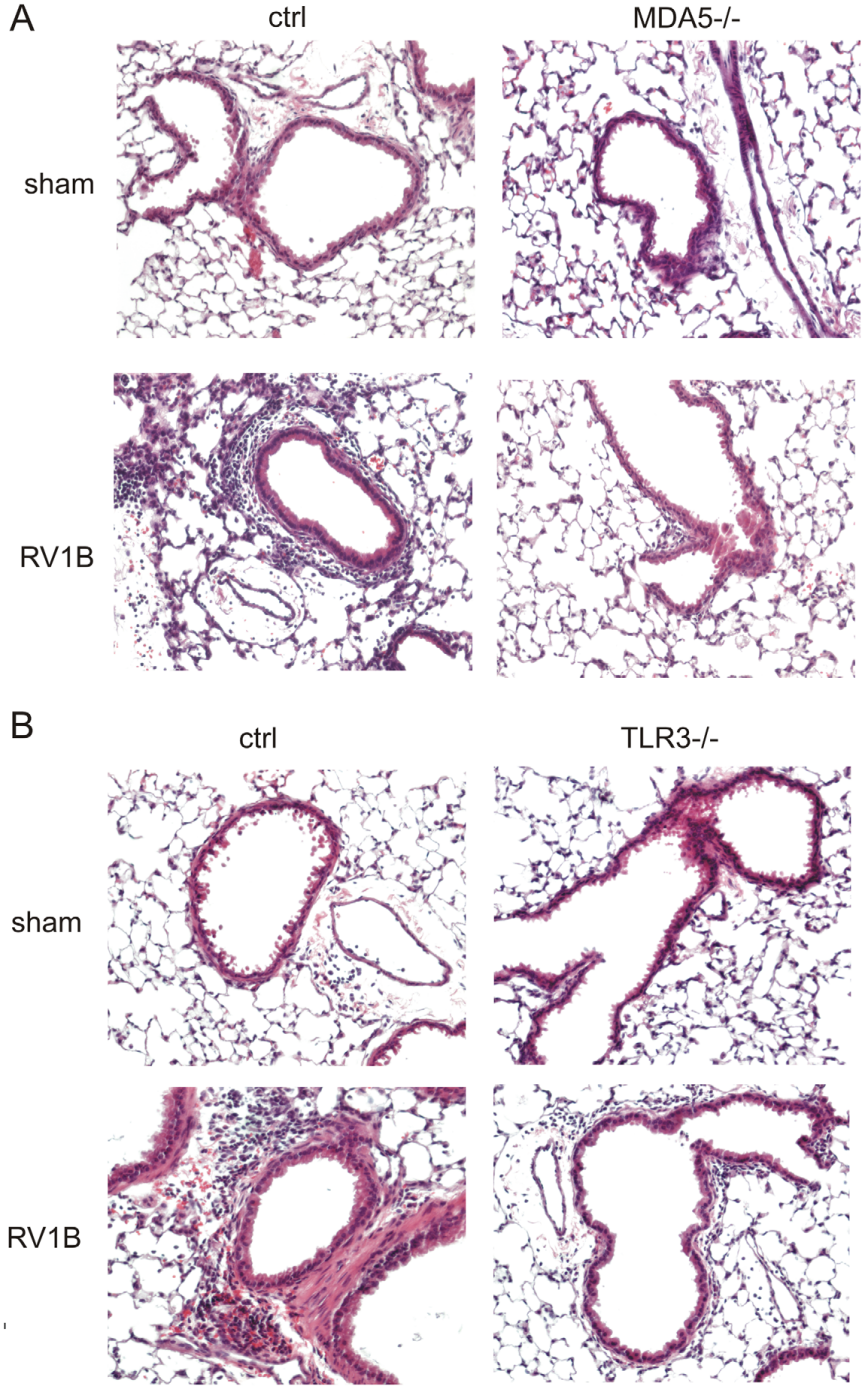
Airway inflammation in RV1B-infected MDA−/− and TLR3−/− mice. MDA5−/− (A), TLR3−/− (B) and their control mice were infected with RV1B. Twenty-four h after infection, lungs were fixed and stained with hematoxylin and eosin (original magnification, 100×).

**Figure 6:**
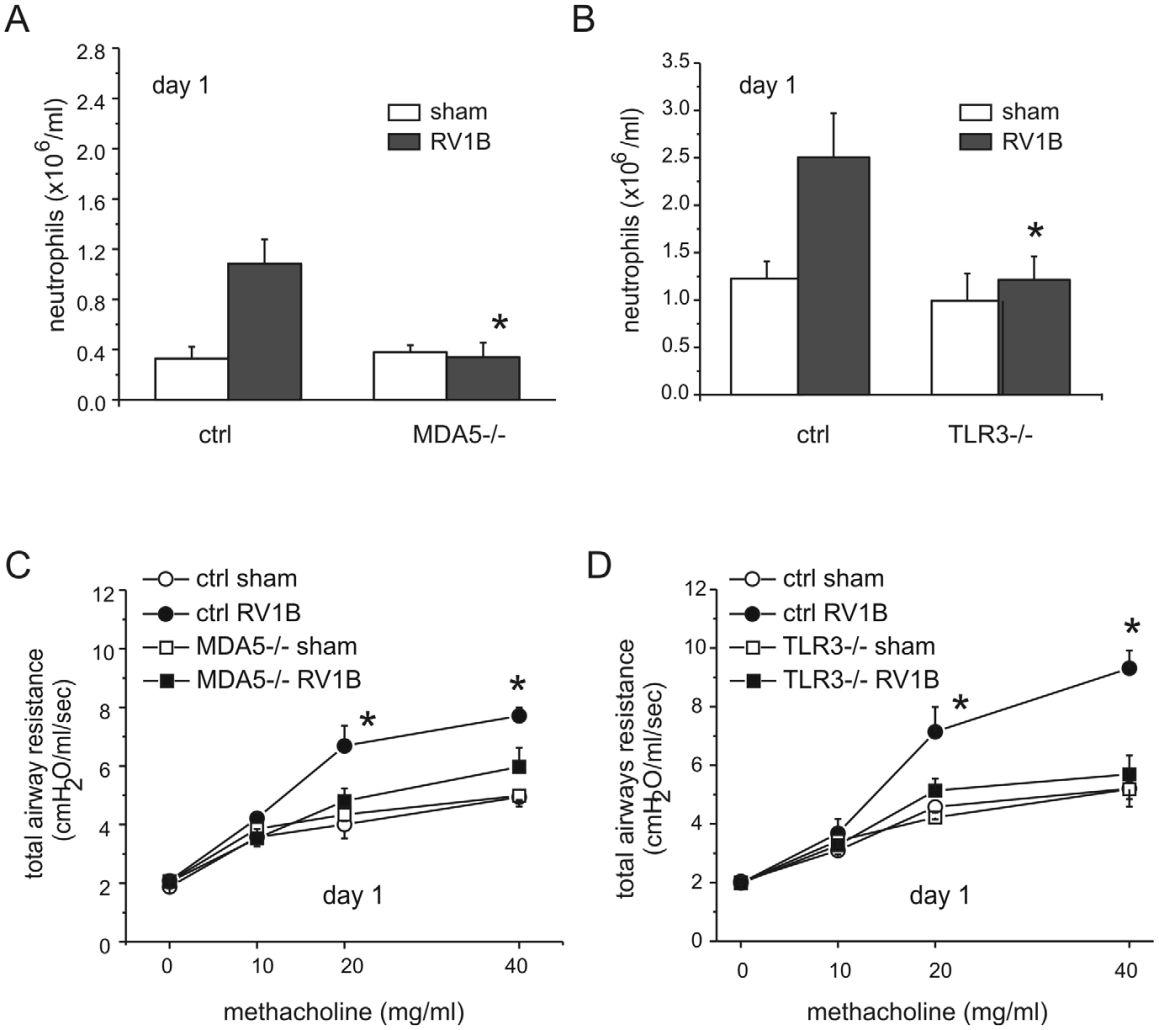
Airway inflammation and responsiveness in RV1B-infected MDA5−/− and TLR3−/− mice. MDA5−/−, TLR3−/−, and their control mice were infected with RV1B. Twenty-four h after infection, RV1B-induced neutrophil infiltration was determined in MDA5−/− mice (A) and TLR3−/− mice (B) along with their respective controls. Data represent mean±SEM for 3–7 mice, *p<0.05, one-way ANOVA). Total respiratory system resistance of MDA5−/− (C) and TLR3−/− (D) mice were determined by plethysmography. Data represent mean±SEM for 3–7 mice (*p<0.05, two-way ANOVA).

To determine whether MDA5 and TLR3 play a role in mediating RV1B-induced inflammatory responses, we measured the expression of pro-inflammatory cytokines in MDA5−/− mice, TLR3−/− and their controls. RV1B-infected MDA5−/− mice and TLR3−/− mice each displayed significantly decreased expression of the neutrophil chemoattractants CXCL1/KC and CXCL2/MIP-2 compared to that of control mice (Figures 3a–d). CXCL1 protein was also decreased (Figures 3e, 3f). To varying degrees, CCL2/MCP-1, CXCL10/IP-10 and eotaxin-1/CCL11 were also decreased in the MDA5 and TLR3 knockout mice (Figure 4a–f). Accordingly, we also found less inflammation in the lungs of RV1B-infected MDA5−/− and TLR3−/− mice compared to control mice 24 h after infection (Figure 5a, 5b). There were significantly fewer neutrophils in the lungs of RV1B-infected MDA5−/− and TLR3−/− mice compared to control mice (Figures 6a, 6b). These data suggest that MDA5 and TLR3 are each required for maximal RV1B-induced inflammatory responses *in vivo*.

### MDA5 and TLR3 are each required for RV1B-induced airway hyperresponsiveness

We have previously shown that RV1B-induced airways cholinergic hyperresponsiveness in naïve (non-allergic) mice is dependent on CXCR2 and airway neutrophilic inflammation [19]. Since RV1B-infected MDA5−/− and TLR3−/− mice each showed significant reductions in lung neutrophils and the CXCR2 ligands CXCL1 and CXCL2, we hypothesized that RV1B-infected knockout mice would show reduced airway responses to methacholine compared to control mice. As noted previously, RV1B-infected control mice displayed significantly higher airways responses to methacholine compared to sham-infected mice (Figures 6c, 6d). However, compared to sham-inoculated mice, RV1B-induced airway responses were not elevated in MDA5−/− and TLR3−/− mice. Together, these results demonstrate that MDA5 and TLR3 signaling initiates pro-inflammatory signaling pathways leading to airways inflammation and hyperresponsiveness. Thus, MDA5- and TLR3-driven innate immune responses to RV are responsible for airway dysfunction in this model.

### MDA5 is required for maximal RV1B-induced type I and III IFN responses in mice with allergic airways disease

**Figure 7:**
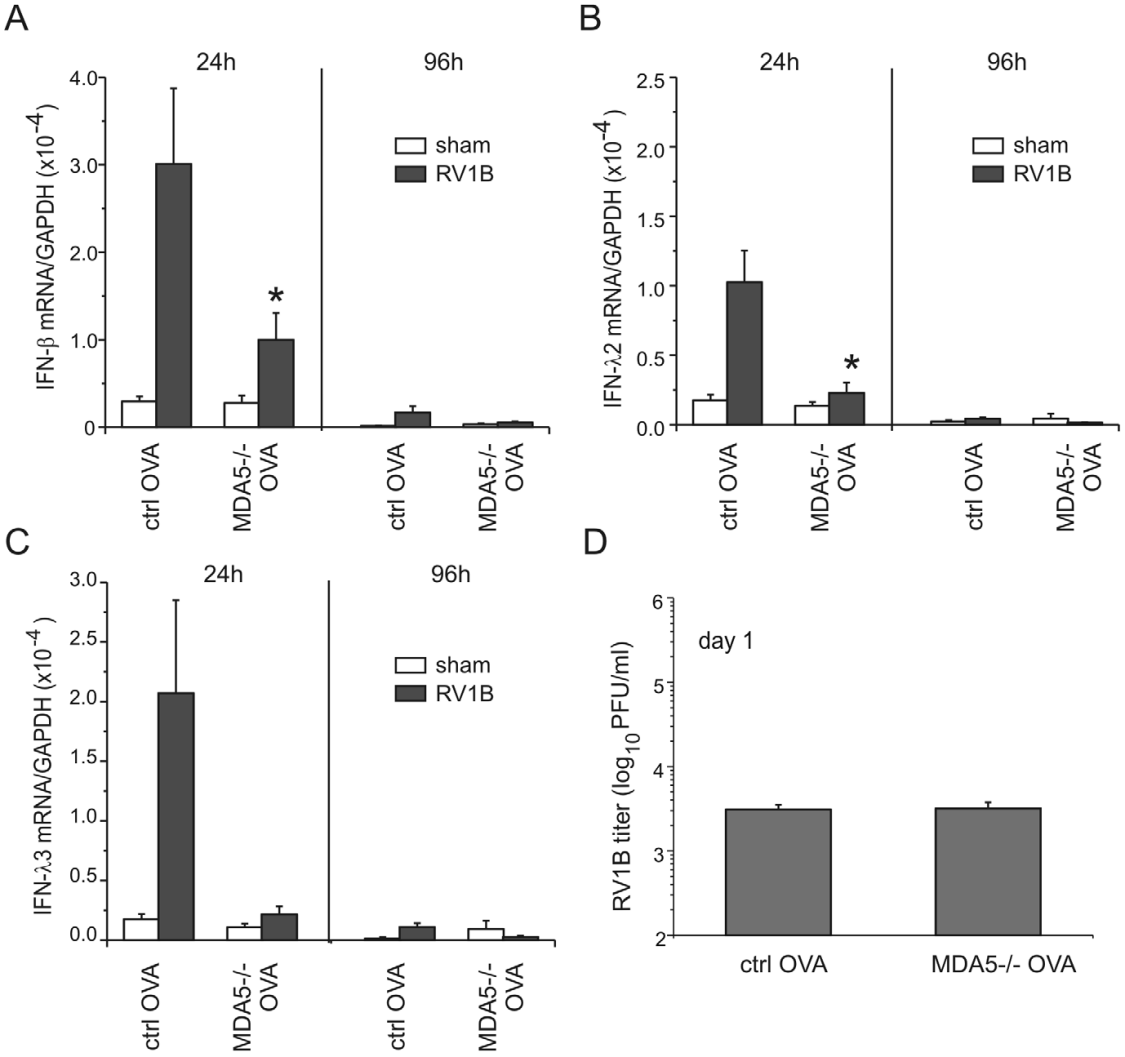
RV1B-induced IFN responses and viral titer in OVA-treated MDA5−/− mice. OVA-treated MDA5−/− mice and control mice were infected with sham or RV1B. Total lungs were harvested at 24 post infection. A–C The expression of IFN-β, IFN-λ2, IFN-λ3 was determined by qPCR. D. Total lung titer at 24 h post infection was determined by plaque assay. The expression of each target gene was normalized to GAPDH. Data represent mean ± SEM for five-seven mice.

Since RV is the most frequent pathogen associated with asthma exacerbations, we sought to examine the effect of MDA5 deficiency, which affected both RV-induced inflammatory and IFN responses, on mice that were sensitized and challenged with ovalbumin (OVA), a commonly-used model of allergic airways disease. Wild-type and MDA5−/− mice were injected intraperitoneally with PBS or a solution of alum and OVA, and then challenged intranasally with PBS or OVA. Mice were infected with RV1B immediately following the last OVA or PBS treatment. Lung inflammatory and IFN responses were measured as previously described in non-allergic, naïve mice. Twenty-four h after infection, RV1B-infected OVA-treated wild-type mice displayed significantly increased levels of IFN-β, IFN-λ2 and IFN-λ3 expression compared to sham-inoculated OVA-treated mice. IFN responses were severely diminished in RV1B-infected OVA-treated MDA5−/− mice (Figures 7a–c). Consistent with our previous results [18], after ovalbumin sensitization and challenge, both control and MDA5−/− mice showed significantly lower viral titers compared to non-allergic mice, indicating either enhanced viral clearance or perhaps a failure of the virus to establish infection (Figure 7d). However, there was no difference observed between OVA-treated control or MDA5−/− mice with respect to viral titers. Thus, in the context of allergic inflammation, MDA5-deficient mice showed reduced viral titers, similar to wild-type mice, despite an abnormal IFN response.

### MDA5 is required for maximal RV1B-induced airways inflammation and hyperresponsiveness in mice with allergic airways disease

**Figure 8:**
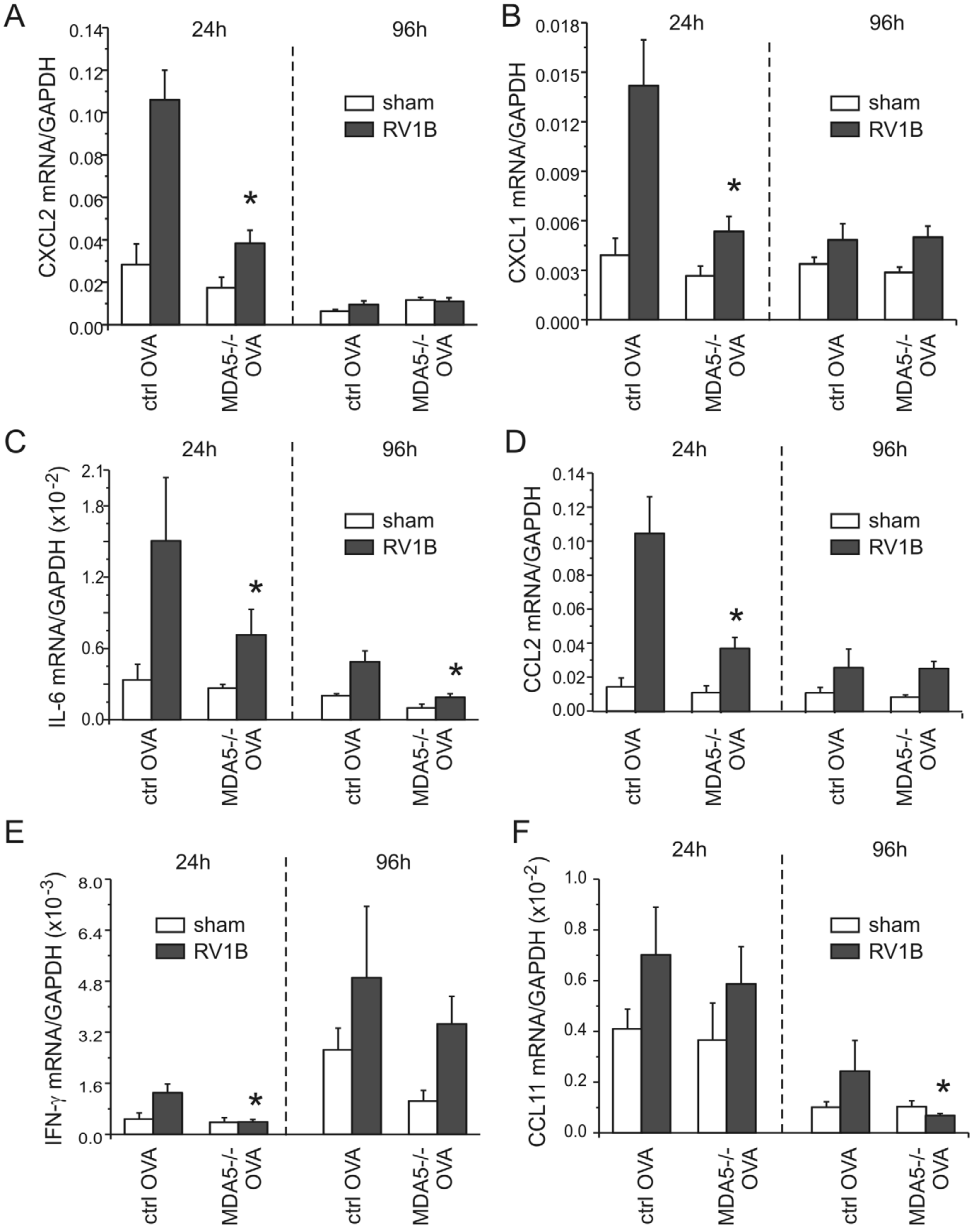
RV1B-induced pro-inflammatory cytokine expression in OVA-treated MDA5−/− mice. OVA-sensitized and -challenged MDA5−/− and control mice were inoculated with sham or RV1B. Lungs were harvested at 24 and 96 h after infection. A–F. The expression of CXCL1/KC, CXCL2/MIP-2, IL-6, CCL2/MCP-1, IFN-γ and CCL11/eotaxin-1 was determined by qPCR. The expression of each target gene was normalized to GAPDH. Data represent mean±SEM for 4–7 mice (*p<0.05, one-way ANOVA).

**Figure 9:**
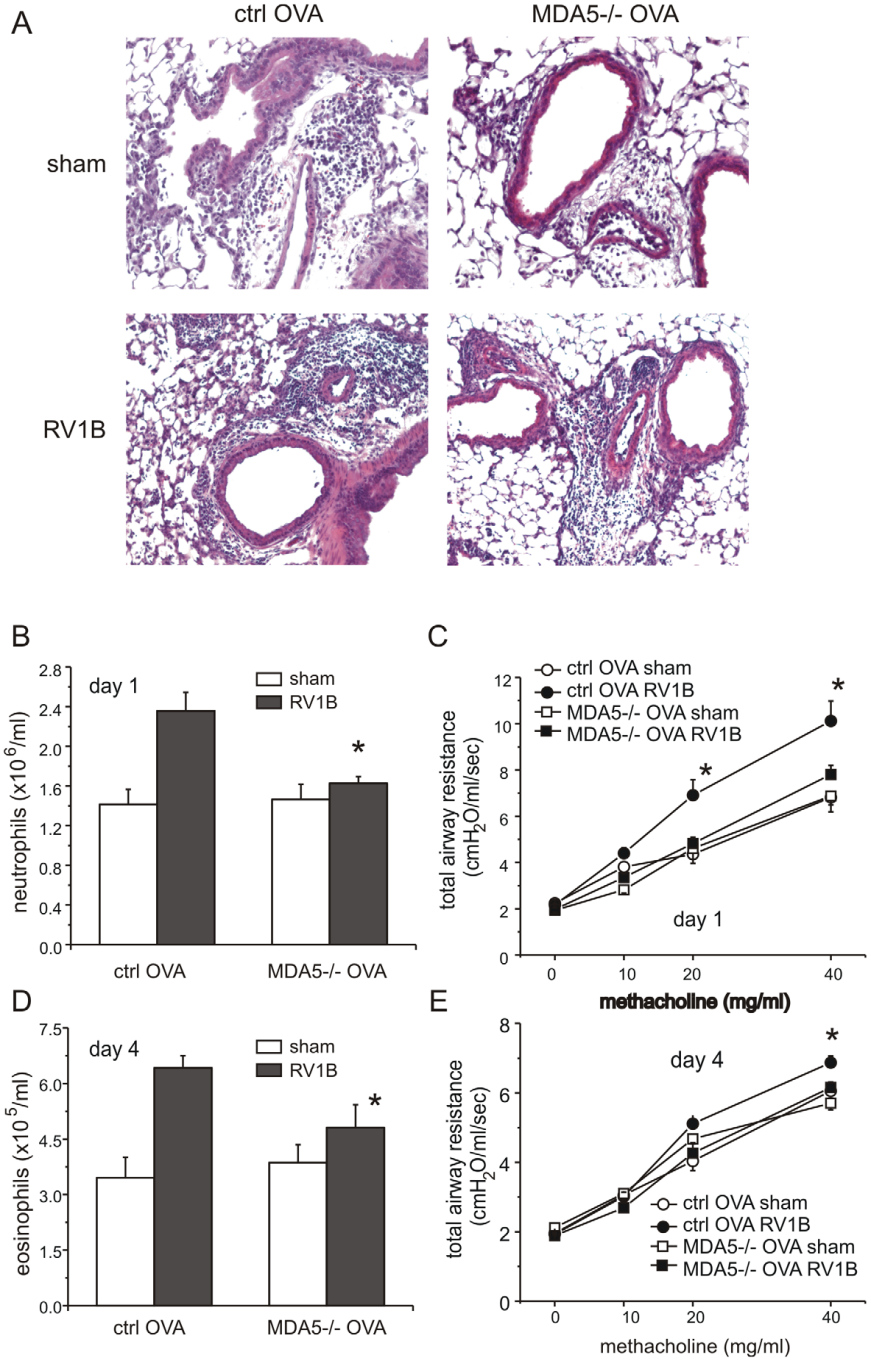
Lung inflammation in OVA-treated RV1B-infected MDA5−/− mice: histology and airways responsiveness. MDA5−/− mice and their control mice were sensitized and challenged with OVA and then infected with RV1B. A. Twenty-four h after infection, lungs were fixed and stained with hematoxylin and eosin (original magnification, 100×). B. Twenty-four h after infection, lungs were digested by collagenase. The number of infiltrated neutrophils were counted. C. Total respiratory system resistance one day after infection was determined by plethysmography. D. Four days after infection, MDA5 null mice showed significantly reduced lung eosinophils. E. Total respiratory system resistance four days after infection. Data represent mean±SEM for 6 mice (*p<0.05, two-way ANOVA).

We have previously shown that RV1B infection and OVA sensitization and challenge have additive effects on lung inflammation in control mice one day after infection [18]. Thus, as expected, sham-infected OVA treated wild-type mice showed more lung inflammation than sham-infected or RV1B-infected naïve wild-type mice. Baseline levels of cytokines (CXCL2/MIP-2, CXCL1/KC, IL-6, CCL2/MCP-1, IFN-γ and CCL11/eotaxin-1) were increased in the lungs of sham-infected OVA-treated mice (Figure 8) compared to the naïve, non-allergic mice (Figures 4a, 4c, S3). Twenty-four h after RV1B infection, CXCL1/KC, CXCL2/MIP-2, IL-6, CCL2/MCP-1 and IFN-γ expression in wild-type mice increased 3–5 fold. MDA5 deficiency significantly reduced the induction of CXCL1/KC, CXCL2/MIP-2, IL-6, CCL2/MCP-1 and IFN-γ. The expression of CCL11/eotaxin-1 mRNA was not significantly elevated 24 h after infection and was not different between control and MDA5−/− mice (Figure 8f). Neutrophil infiltration in the MDA5−/− mice was significantly lower than wild-type mice (Figures 9a, 9b). Hematoxylin and eosin staining showed less inflammation around the airways of RV1B-infected OVA-treated MDA5−/− mice compared to RV-infected OVA-treated wild-type mice. In contrast, there was no significant difference observed in the number of macrophages, eosinophils or lymphocytes (Figure S4). Consistent with the greater amount of inflammation present in the lung, RV1B-infected, OVA-treated control mice displayed the highest airways responsiveness compared to any other treatment, and MDA5 deficiency essentially eliminated this hyperresponsiveness (Figure 9c).

Ninety-six h after infection, RV1B-induced airways hyperresponsiveness is dependent on CCL11/eotaxin-1-mediated eosinophilic airway inflammation [18]. At this time point, in contrast to the other cytokines, IFN-γ mRNA expression increased in both control and MDA5−/− mice (Figures 8e). Also, compared to control mice, RV1B-induced IL-6 and CCL11/eotaxin-1 levels were significantly decreased in MDA5−/− mice (Figures 8c, 8f). No differences in neutrophil, lymphocyte or macrophage infiltration between wild-type and MDA5 null mice were observed (Figure S5). However, eosinophil infiltration was significantly lower in MDA5−/− mice (Figure 9d). Finally, the airways responsiveness of RV1B-infected OVA-treated MDA5−/− mice was decreased at 96 h post-infection (Figure 9e).

### TLR3 is required for maximal RV1B-induced airways responses in mice with allergic airways disease

**Figure 10:**
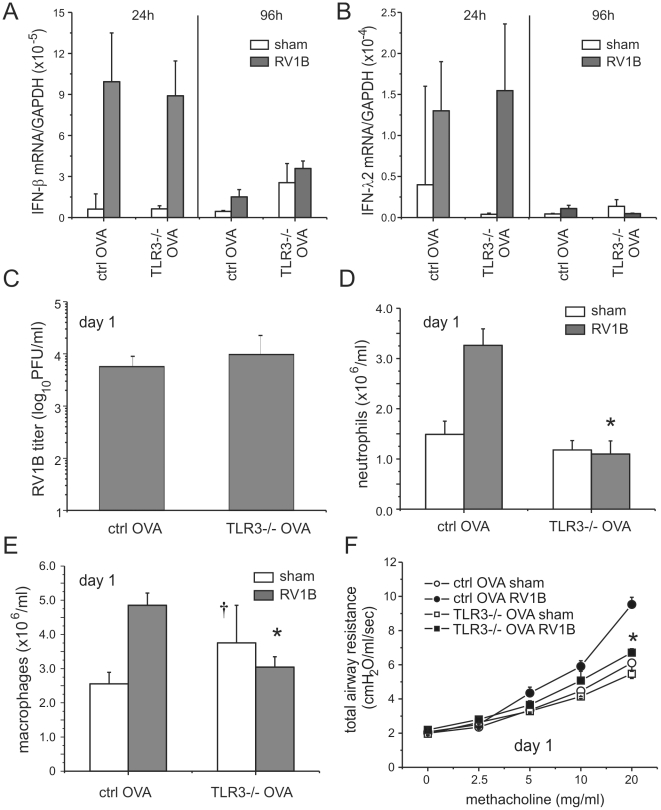
Airway responses in RV1B-infected, OVA-sensitized and -challenged TLR3 null mice. A, B. mRNA expression of IFN-β and IFN-λ2 24 and 96 h after infection was determined by qPCR. The expression of each target gene was normalized to GAPDH. C. Viral titer measured 24 h after RV1B infection of allergic wild-type and TLR3−/− mice. D, E. Lungs were digested by collagenase and the number of infiltrated neutrophils and macrophages were counted. F. Total respiratory system resistance one day after infection was determined by plethysmography. Data represent mean±SEM for 4 mice (*different from wild-type RV1B-infected mice, p<0.05, one- or two-way ANOVA; †different from sham-inoculated mice, p<0.05, one-way ANOVA).

We also tested the effects of TLR3 knockout on RV1B-induced airways inflammation and responsiveness in OVA-sensitized and -challenged mice. As with mice unexposed to OVA, there was no effect of TLR3 knockout on RV1B-induced IFN-β, IFN-λ2 or IFN-λ3 mRNA levels (Figures 10a, 10b, S6). As in our experiments with MDA5 null and control mice, OVA sensitization and challenge was associated with a reduction in viral titer (Figure 10c). RV1B-induced infiltration of neutrophils and macrophages into the lungs of OVA-treated TLR3−/− mice was significantly lower than wild-type mice (Figures 10d, 10e). There was no effect of TLR3 knockout on day 1 lymphocytes or eosinophils (Figure S6) or day 4 cell counts (not shown). RV1B-infected, OVA-sensitized and -challenged mice with TLR3 deficiency demonstrated significantly lower airways responsiveness to inhaled methacholine one day after infection than similarly-infected and -treated wild-type mice (Figure 10f). By day 4, there was no difference in airways responsiveness between groups (Figure S6).

**Figure 11:**
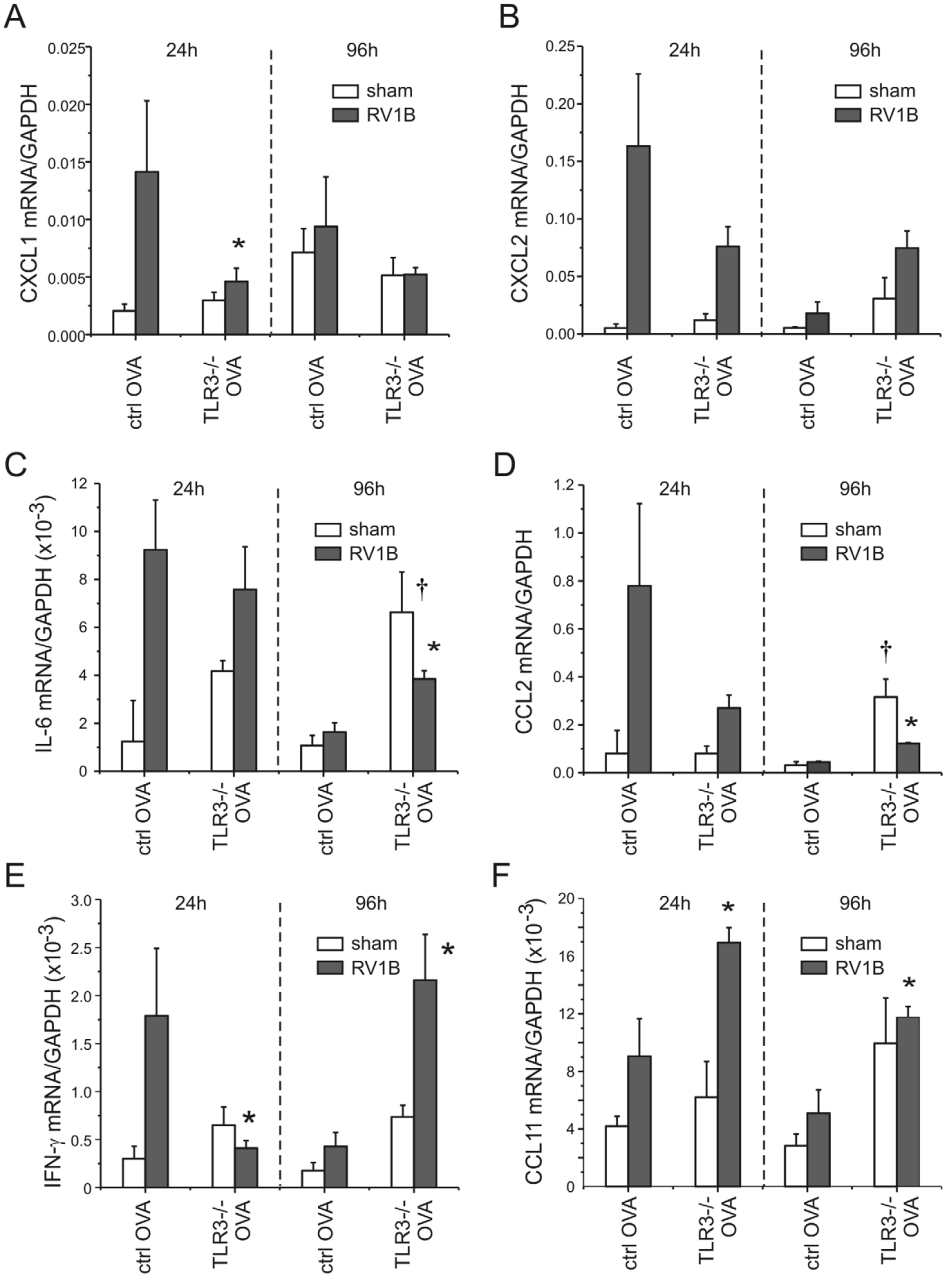
RV1B-induced pro-inflammatory cytokine expression in OVA-treated TLR3−/− mice. OVA-sensitized and -challenged TLR3−/− and control mice were inoculated with sham or RV1B. Lungs were harvested at 24 and 96 h after infection. A–F. The expression of CXCL1/KC, CXCL2/MIP-2, IL-6, CCL2/MCP-1, IFN-γ and CCL11/eotaxin-1 was determined by qPCR. The expression of each target gene was normalized to GAPDH. Data represent mean±SEM for 4 mice (*different from wild-type RV1B-infected mice, p<0.05, one-way ANOVA; †different from sham-inoculated mice, p<0.05, one-way ANOVA).

Consistent with the lower number of neutrophils in the lungs of TLR3 knockout mice, mRNA expression of the neutrophil chemokine CXCL1/KC was decreased one day after RV1B infection (Figure 11a). CXCL2, CCL2 and IFN-λ levels were also decreased to varying degrees one day after infection (Figure 11c–e). Together, these data suggest that, in mice with allergic airways disease, TLR3 signaling initiates pro-inflammatory responses which augment airways cholinergic responsiveness. On the other hand, four days after infection, TLR3−/− mice showed significantly increased mRNA expression of IL-6, CCL2, CCL11 and IFN-λ (Figures 11c–f).

## Discussion

Pattern recognition receptors regulate multiple effector molecules, including type I IFNs and other pro-inflammatory cytokines [20], [21], [22]. The innate immune response to viral infection is specific to cell type/organ as well as to invading pathogen [23], [24]. The ultimate host response is likely to be an integration of both IFN and pro-inflammatory responses. Because viral infections, in particular RV, are the most common cause of asthma exacerbations, we examined the roles of the pattern recognition receptors TLR3 and MDA5 in the response to RV infection. We found that both TLR3 and MDA5 were required for RV1B-induced maximal inflammatory responses and airways cholinergic hyperresponsiveness *in vivo*. MDA5 mice also showed a delayed type I IFN and attenuated type III IFN response to RV1B infection, leading to a transient increase in viral titer. Further, MDA5 null mice with allergic airways disease showed reduced viral titers compared to non-allergic mice despite deficient IFN responses, and allergic MDA5 and TLR3 null mice each showed decreased RV-induced airway inflammatory and contractile responses. Together, these results suggest that TLR3 and MDA5 individually initiate pro-inflammatory signaling pathways leading to airways inflammation and cholinergic hyperresponsiveness.

Mice lacking TLRs have been shown to display diverse viral infectious phenotypes depending on the host gene-pathogen combination. Studies have shown that TLR3 plays a protective role against various viral infections *in vivo*. Mice lacking TLR3 display reduced IFN-α/β production and an increased viral load against mouse cytomegalovirus infection [25]. In response to coxsackievirus B4 infection, TLR3−/− mice produce less pro-inflammatory mediators and are unable to control viral replication at the early stages of infection, resulting in severe cardiac damage and reduced survival [26]. Compared to wild-type mice, TLR3−/− mice show increased airway mucus, airways responsiveness and IL-13 expression with RSV infection [27], suggesting that TLR3 signaling may be protective against airway pathology with this virus. On the other hand, other studies have suggested that TLR3 plays a detrimental role upon viral infection. Despite an increased viral load, influenza A virus-infected TLR3−/− animals display significantly reduced pro-inflammatory mediators, suggesting that TLR3 critically contributes to a detrimental host inflammatory response [28]. In addition, TLR3 signaling has also been reported to have a detrimental effect in phlebovirus and vaccinia infections [29], [30]. TRIF−/− mice challenged with dsRNA display decreased airway responsiveness and pulmonary inflammation compared to control mice [31]. The precise role of TLR3 in West Nile virus infections is uncertain [32], [33]. Our study demonstrated that, as with influenza infection, TLR3−/− mice display decreased airway neutrophilic inflammation upon RV1B infection, resulting in a significantly lower airways cholinergic responsiveness, suggesting for the first time that TLR3 signaling is responsible for airway dysfunction following RV infection.

In contrast to the effect of TLR3 deficiency on airways inflammation, TLR3 null mice showed normal IFN production. However, MDA5−/− mice displayed a reduction in type I IFN expression after RV1B infection, leading to a transient increase in viral load and copy number 24 h after infection. MDA5−/− mice displayed increased IFN-α/β expression 48 h after RV1B infection, suggesting a delayed onset of IFN signaling. We speculate that this was due to either the compensatory activation of another host pattern recognition receptor, or the dysregulation of normal innate immune system activation in the absence of MDA5-mediated signaling. On the other hand, MDA5 null mice showed a persistent defect in the expression of type III IFNs. These data suggest that type III IFN responses are a less critical host defense against RV1B infection and are regulated independently of type I IFN responses. In support of this concept, IFN-λ is not required for immunity to influenza in wild-type mice, though it protects influenza-infected IFN-α/β knockout mice [34], suggesting that type III IFNs play a secondary and redundant antiviral role in innate immunity. Together, these data suggest that MDA5 rather than TLR3 is the primary receptor responsible for the initial IFN response to RV infection, and that type I IFNs play a role in the containment of RV infection.

In addition to decreased IFN responses, MDA5−/− mice also displayed reduced expression of pro-inflammatory cytokines such as CXCL1, CXCL2, CCL2 and IL-6. The coupling of IFN and pro-inflammatory responses in MDA5-deficient mice has been observed in other studies. Bone marrow-derived dendritic cells from MDA5−/− mice display significantly lower expression of IFN-α, CCL2/MCP-1 and IL-6 against murine norovirus infection compared those from control mice [23]. On the other hand, reduced viral clearance has ultimately led to enhanced inflammatory responses in some models. MDA5−/− mice show significantly decreased IFN mRNA expression profiles five days after Sendai virus infection but compensatory IL-6 mRNA expression, resulting in increased mortality and severe histopathological changes in the lower airways [35]. Thus, host inflammatory responses in the absence of IFN expression are pathogen-specific: When challenged by pathogenic lethal viruses such as Sendai virus, the host initiates inflammatory responses to defend against viral invasion. However, when confronted with non-pathogenic viruses such as RV, it may be advantageous for the host to terminate the inflammatory response, in order to avoid adverse effects.

It has been proposed that asthmatics are susceptible to RV infection due to deficient IFN production. RV-infected airway epithelial cells from asthmatic subjects show impaired production of IFN-β and -λ [13], [14] and asthmatics experimentally infected with RV16 showed a reduced IFN-γ/IL-5 mRNA ratio in their sputum [15]. To examine whether an allergic background alters the response of wild-type and MDA5 knockout mice to viral infection, we combined RV infection with a commonly-used model of allergic airways disease, OVA-sensitization and challenge. First, we found that, following OVA treatment, both wild-type and MDA5−/− mice demonstrated reduced viral titers following infection with RV. MDA5 null mice showed reduced viral titers despite attenuated IFN responses. This finding is consistent with our previous data [18], as well as data from guinea pigs that were sensitized to OVA and subsequently infected with parainfluenza [36]. The precise mechanism for decreased viral titers in mice with allergic airways disease is unclear. Prior to RV infection, these mice showed increased baseline levels of neutrophils, macrophages, lymphocytes, eosinophils, IFN-γ, IL-6 and CCL11/eotaxin-1, each of which could have contributed to an antiviral response. Eosinophils are known to contain granule proteins such as eosinophil cationic protein and eosinophil-derived neurotoxin,which possess strong ribonuclease activity that can neutralize viruses [37]. Eosinophils have been shown to neutralize RSV in a concentration-dependent manner, and this effect could be completely reversed by a ribonuclease inhibitor [38], [39]. Another possible mediator is nitric oxide, an antiviral which is produced by epithelial cells in response to RV infection [40]. Whatever the mechanism, our finding of reduced viral titers in MDA5−/− mice with attenuated IFN responses demonstrates that other factors besides IFNs may play a role in the containment and clearance of RV infection.

Second, we found that RV1B-infected OVA-treated MDA5−/− mice showed significantly reduced IFN and cytokine levels compared to wild-type mice. Reduced cytokine expression, in turn, led to persistent attenuations in airway inflammation and responsiveness. Thus, pro-inflammatory and IFN responses were strictly linked: Reduced IFN responses in MDA5−/− mice were associated with less robust, not increased, inflammatory responses. These data are consistent with our recent findings in airway epithelial cells isolated from patients with COPD [41]. These cells showed increases in both pro-inflammatory cytokines and IFNs.

In general, TLR3-deficient mice with allergic airways disease showed similarly attenuated inflammatory responses to RV1B infection, leading to reduced airways responsiveness. However, there were some differences between MDA5 and TLR3 null mice. As with non-allergic mice, TLR3 knockout mice showed normal IFN responses to RV infection. In addition, TLR3 mice showed higher levels of some pro-inflammatory cytokines four days after infection, consistent with compensatory activation of another host pattern recognition receptors. On the other hand, a recent study showed that administration of synthetic dsRNA attenuates allergic inflammation [42], suggesting that TLR3 signaling may also have anti-inflammatory effects. Finally, disparities between MDA5 and TLR3 null mice be attributed to differences in genetic background.

Finally, we would like to add a few comments about our mouse model of human RV1B infection. We [17] and others [43] have found that a much higher viral titer is required to infect mice compared to humans. This is to be expected, as differences in the homology of viral receptors and intracellular signaling mechanisms are likely to restrict viral infection and replication in mice. This restriction in viral replication could have limited the effects of pattern recognition receptor knockout in our model. For example, had viral replication been more robust, it is conceivable that TLR3 and MDA5 knockout could have led to more severe airways disease, rather than the attenuated responses we observed. On the other hand, we suspect our results are physiologic, for the following reasons: First, viral replication in the lungs of humans is likely to be small. Despite the fact that RV is a well-established cause of asthma and COPD exacerbations in humans, it has rarely been cultured from the lower airways of non-immunosuppressed patients. Second, RV causes minimal cytotoxicity in infected epithelial cells. Whatever the level of viral replication, our results demonstrate that engagement of TLR3 and MDA5 by RV dsRNA initiates pro-inflammatory signaling pathways leading to airways inflammation and cholinergic hyperresponsiveness. To our knowledge this is the first study to examine the contribution of TLR3 and MDA5 to RV responses *in vivo*.

Our data raise the possibility that TLR3- and MDA5-driven innate immune responses to RV, a relatively non-pathogenic virus, are maladaptive, at least at the onset of infection. If this were true in humans, then antagonists against TLR3 and MDA5 could provide potential therapeutic agents in the treatment of virus-induced asthma exacerbations. Future studies focusing on the interactions and coordination between the two receptors would be useful in understanding the precise mechanism of RV-induced, pattern recognition receptor-mediated innate immune responses.

## Materials and Methods

### Ethics statement

This study was carried out in strict accordance with the recommendations in the Guide for the Care and Use of Laboratory Animals of the National Institutes of Health. The protocol was approved by the Institutional Animal Care and Use Committee of the University of Michigan Medical School. All surgery was performed under sodium pentobarbital anesthesia, and all efforts were made to minimize suffering.

### Animals

A breeding pair of MDA5−/− mice, developed on a >99.5% C57BL/6 background, was generously provided by Dr. Marco Colonna (Washington University, St. Louis) [23]. Mice were then bred at the University of Michigan animal facility. Control C57BL/6J mice were purchased from Jackson Laboratories. TLR3−/− and B6;129SF2/J control mice were also purchased from Jackson Laboratories (Bar Harbor, MA). Six-to-eight week old female mice were used in this study. All mice were housed in a specific pathogen-free area within the animal care facility.

### Generation of RV and titer determination

RV1B (ATCC, Manassas, VA) was concentrated, purified and titered as described previously [44], [45]. Viral titer was determined by plaque assay [46]. To produce replication-deficient virus, RV1B was UV-irradiated using a CL-1000 crosslinker (UVP, Upland, CA).

### RV exposure and ovalbumin (OVA) sensitization/challenge

Mice were inoculated intranasally with 45 µl of 1×10^8^ TCID_50_/ml RV1B, UV-irradiated RV or an equal volume sham HeLa cell lysate [17]. For OVA sensitization, mice were injected intraperitoneally on days 1 and 7 with 0.2 ml PBS or a solution of alum and 100 µg endotoxin-free OVA (Sigma-Aldrich, St. Louis, MO). Next, mice were challenged intranasally with 50 µl of PBS or 100 µg OVA on days 14, 15 and 16. Selected mice were infected with RV1B immediately following the last OVA or PBS treatment.

### Lung inflammation

To quantify inflammatory cells, lung digests were obtained by mincing the tissue, proteolysis in collagenase type IV (Gibco Invitrogen, Carlsbad, CA) and straining through a 70 µm nylon mesh (BD Falcon, San Jose, CA), as described [47]. The resulting pellet was treated with red blood cell lysis buffer (BD Pharmingen, San Diego, CA) and leukocytes were enriched by spinning the cells through 40% Percoll (Sigma-Aldrich). The total cell count was determined on a hemocytometer. Cytospins were performed from lung digested leukocytes and were then stained by Diff-Quick method (Dade Behring, Newark, DE). Differential counts were determined by counting 200 cells per slide.

### Measurement of airways responsiveness

Mice were anesthetized with sodium pentobarbital (50 mg/kg mouse, intraperitoneal injection) and a tracheostomy performed. Mechanical ventilation was performed and total respiratory system measured using a Buxco FinePointe operating system (Wilmington, NC). Airway responsiveness was assessed by measuring changes in resistance in response to increasing doses of nebulized methacholine, as described [17].

### Histology

Lungs were fixed in 10% formalin at an inflation pressure of 30 cmH_2_O overnight, transferred to 70% ethanol and paraffin embedded. Five μ sections were stained with hematoxylin and eosin.

### Cytokine/chemokine expression

Total lung RNA was extracted using the RNeasy kit (Qiagen, Alameda, CA) and then transcibed to first-strand cDNA using Taqman reverse transcription reagents (Applied Biosystems Life Technologies, Carlsbad, CA). First-strand cDNA was then used to quantify the expression of IFN-α, IFN-β, IFN-λ2, IFN-λ3, IFN response factor (IRF)-7, CXCL10/IP-10, IFN-γ, CXCL1/KC, CXCL2/MIP-2, CCL2/MCP-1, and CCL11/eotaxin-1 by quantitative two-step real time PCR using specific Syber green primers. All primers were designed and purchased from IDT (Coralville, IA).

### Cytokine production

Lungs were homogenized in 1 ml PBS with protease inhibitor cocktail, spun for 15 minutes at 1500 g, and the supernatant assayed for CXCL1, CXCL2 and IFN-β by ELISA (R&D Systems, Minneapolis, MN; PBL InterferonSource, Piscataway, NJ).

### Presence of viral RNA

RNA was extracted from lungs of mice using Trizol reagent (Sigma-Aldrich, St. Louis, MO) and analyzed for the presence of viral RNA by reverse transcriptase-PCR. Quantitative one-step real time PCR for positive-strand viral RNA was conducted using RV-specific primers and probes (forward primer: 5′-GTG AAG AGC CSC RTG TGC T-3′; reverse primer: 5′-GCT SCA GGG TTA AGG TTA GCC-3′; probe: 5′-FAM-TGA GTC CTC CGG CCC CTG AAT G-TAMRA-3′ [14]. Copy numbers of positive strand viral RNA were normalized to 18S RNA, which was similarly amplified using gene-specific primers and probes.

### Data analysis

Mice from each experimental group were studied in 2–4 separate experiments. SigmaStat computing software (SPSS, Chicago, IL) was used for data analysis. Data are represented as mean±SEM. Statistical significance was assessed by one- or two-way analysis of variance (ANOVA). Differences identified by ANOVA were pinpointed by the Student Newman-Keuls' multiple range test.

## Supporting Information

Figure S1 RV1B-induced expression of type I and III IFNs in TLR3−/− mice. TLR3−/− and their control mice were inoculated with sham, UV-irradiated RV1B (UV RV1B) or intact RV1B. Lungs were harvested at 4, 24, 48, and 96 h after infection. A–D. The expression of IFN-α, IFN-β, IFN-λ2 and IFN-λ3 at each time point was determined by qPCR. E. IFN-β protein production was measured by ELISA at 24 h post-infection. The expression of each target gene was normalized to GAPDH. Data represent mean±SEM for 3–7 mice.
(TIF)

Figure S2 RV1B titers and viral RNA levels in control and TLR3−/− mice. TLR3−/− and their control mice were infected with RV1B. Lungs were harvested at 4, 24, 48, and 96 h post infection. A. Total lung titer at 24 h post-infection was determined by plaque assay. B. RV1B copy number at each time point was determined by qPCR. RV copy number was normalized to 18S rRNA. Data represent mean±SEM for 3–7 mice.
(TIF)

Figure S3 RV1B-induced IL-6 and IFN-γ expression in MDA5−/− mice. MDA5−/− and their control mice were inoculated with sham, UV-irradiated RV1B (UV RV1B) or RV1B. Lungs were harvested 24 after infection. A–B. The expression of IL-6 and IFN-γ were determined by qPCR. The expression of each target gene was normalized to GAPDH. Data represent mean±SEM for 3–7 mice.
(TIF)

Figure S4 Early lung macrophage, eosinophil and lymphocyte counts in OVA-treated RV1B-infected MDA5−/− mice. MDA5−/− mice and their control mice were sensitized and challenged with OVA and then infected with RV1B. Twenty-four h after infection, lungs were digested by collagenase. A–C. The numbers of infiltrated macrophages, eosinophils, lymphocytes were counted. Data represent mean±SEM for 6 mice.
(TIF)

Figure S5 Late lung neutrophil, lymphocyte and macrophage counts in OVA-treated RV1B-infected MDA5−/− mice: histology and airways responsiveness. MDA5−/− mice and their control mice were sensitized and challenged with OVA and then infected with RV1B. Ninety-six h after infection, lungs were digested by collagenase. A–C. The numbers of infiltrated neutrophils, lymphocytes, and macrophages were counted. Data represent mean±SEM for 4–7 mice.
(TIF)

Figure S6 Cell counts, IFN-l3 mRNA levels and late airways responsiveness in OVA-treated RV1B-infected TLR3−/− mice. TLR3−/− mice and their control mice were sensitized and challenged with OVA and then infected with RV1B. A, B. Twenty-four h after infection, lungs were digested by collagenase and the numbers of infiltrated lymphocytes and eosinophils counted. C. The mRNA expression of IFN-λ3 was determined by qPCR. Expression was normalized to GAPDH. D. Airway cholinergic responsiveness 96 h after RV1B infection. Data represent mean±SEM for 4 mice.
(TIF)
