# Supplementary figures and images for: MDA5 and TLR3 Initiate Pro-Inflammatory Signaling Pathways Leading to Rhinovirus-Induced Airways Inflammation and Hyperresponsiveness
Source: PLoS Pathog. 2011 May 26;7(5):e1002070. doi: 10.1371/journal.ppat.1002070 (PMC3102730; doi:10.1371/journal.ppat.1002070)

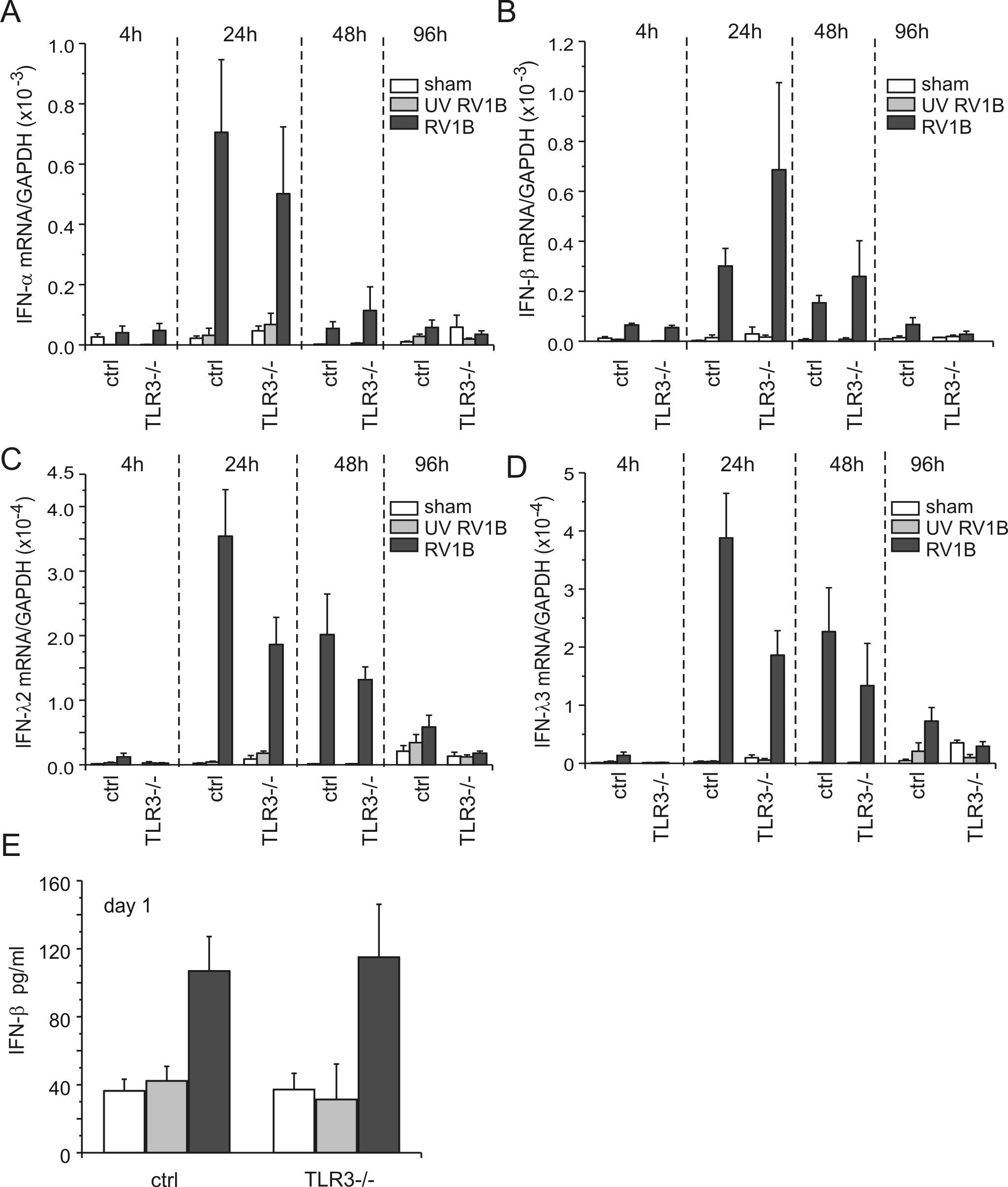

Supplement: Figure S1 — RV1B-induced expression of type I and III IFNs in TLR3−/− mice. TLR3−/− and their control mice were inoculated with sham, UV-irradiated RV1B (UV RV1B) or intact RV1B. Lungs were harvested at 4, 24, 48, and 96 h after infection. A–D. The expression of IFN-α, IFN-β, IFN-λ2 and IFN-λ3 at each time point was determined by qPCR. E. IFN-β protein production was measured by ELISA at 24 h post-infection. The expression of each target gene was normalized to GAPDH. Data represent mean±SEM for 3–7 mice. (TIF) [file ppat.1002070.s001.tif]

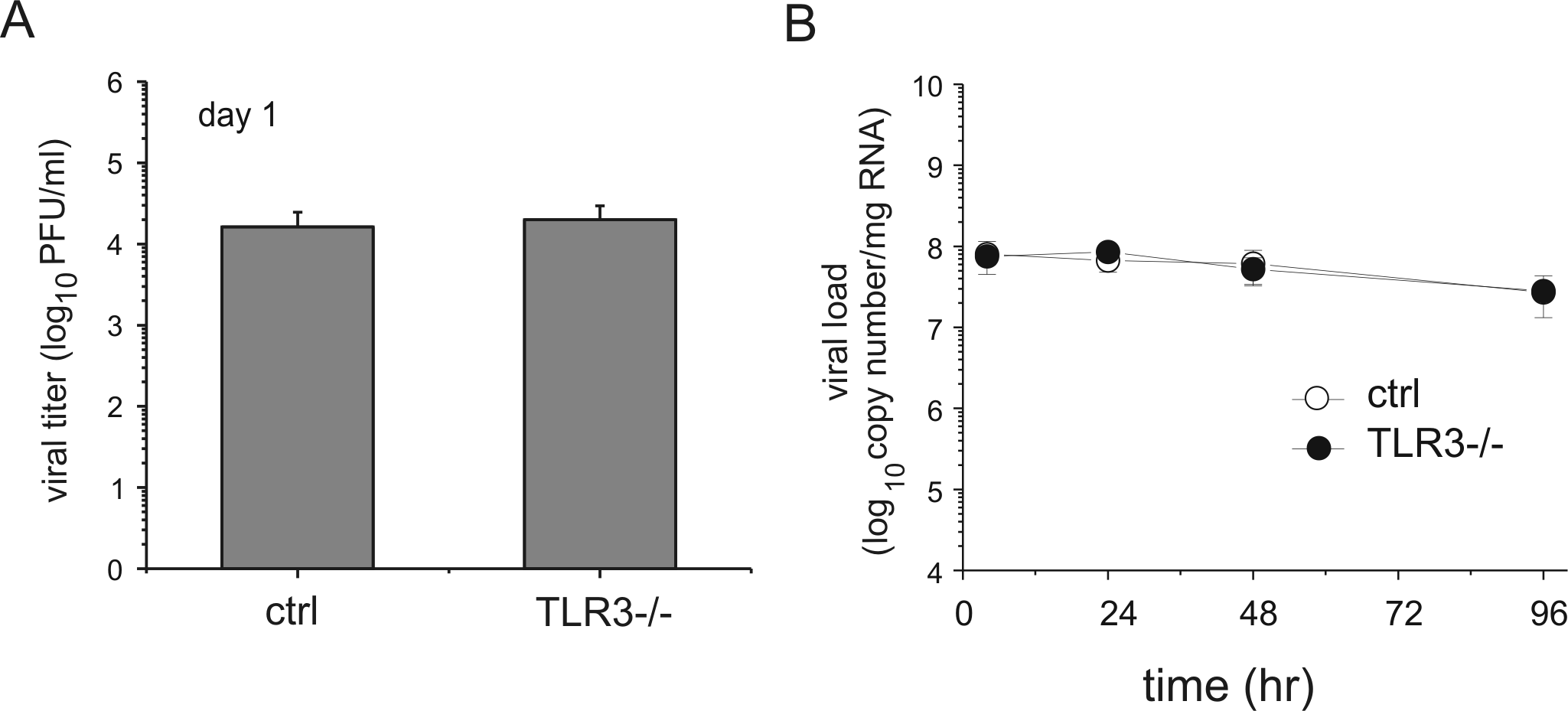

Supplement: Figure S2 — RV1B titers and viral RNA levels in control and TLR3−/− mice. TLR3−/− and their control mice were infected with RV1B. Lungs were harvested at 4, 24, 48, and 96 h post infection. A. Total lung titer at 24 h post-infection was determined by plaque assay. B. RV1B copy number at each time point was determined by qPCR. RV copy number was normalized to 18S rRNA. Data represent mean±SEM for 3–7 mice. (TIF) [file ppat.1002070.s002.tif]

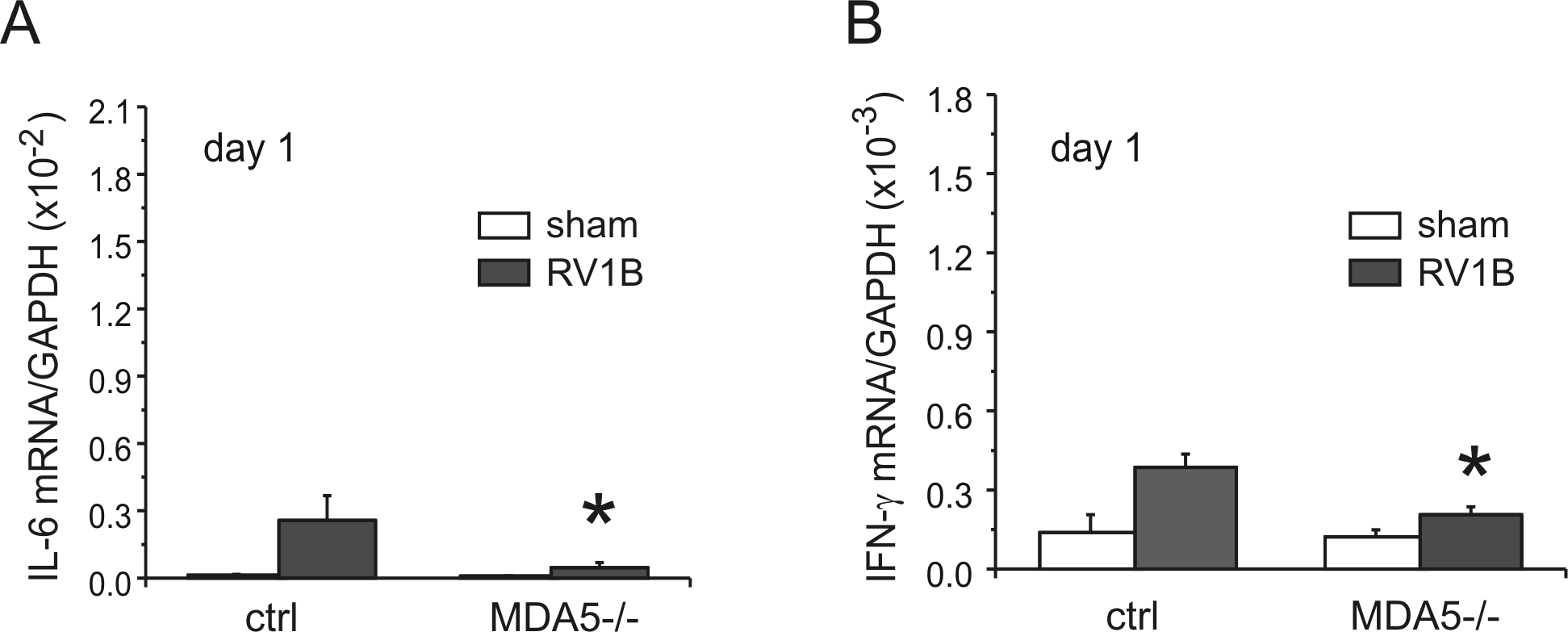

Supplement: Figure S3 — RV1B-induced IL-6 and IFN-γ expression in MDA5−/− mice. MDA5−/− and their control mice were inoculated with sham, UV-irradiated RV1B (UV RV1B) or RV1B. Lungs were harvested 24 after infection. A–B. The expression of IL-6 and IFN-γ were determined by qPCR. The expression of each target gene was normalized to GAPDH. Data represent mean±SEM for 3–7 mice. (TIF) [file ppat.1002070.s003.tif]

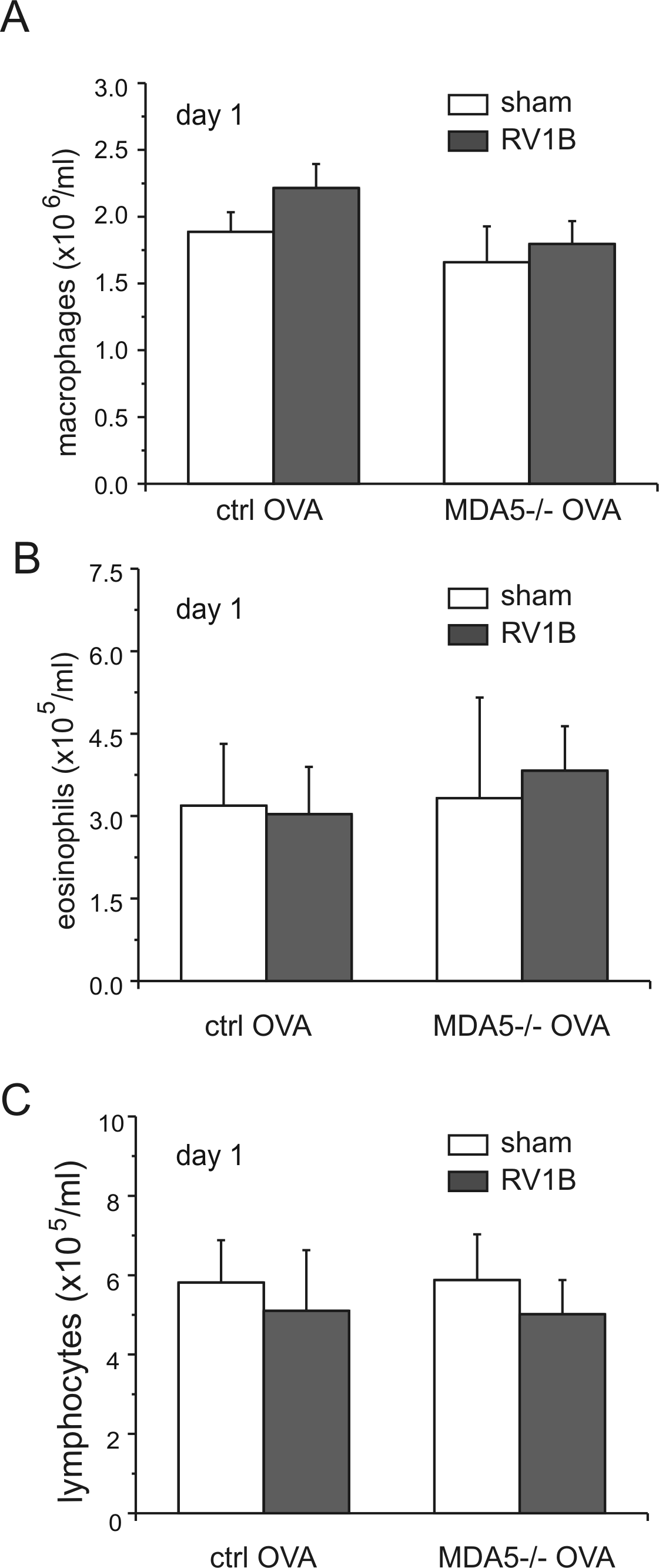

Supplement: Figure S4 — Early lung macrophage, eosinophil and lymphocyte counts in OVA-treated RV1B-infected MDA5−/− mice. MDA5−/− mice and their control mice were sensitized and challenged with OVA and then infected with RV1B. Twenty-four h after infection, lungs were digested by collagenase. A–C. The numbers of infiltrated macrophages, eosinophils, lymphocytes were counted. Data represent mean±SEM for 6 mice. (TIF) [file ppat.1002070.s004.tif]

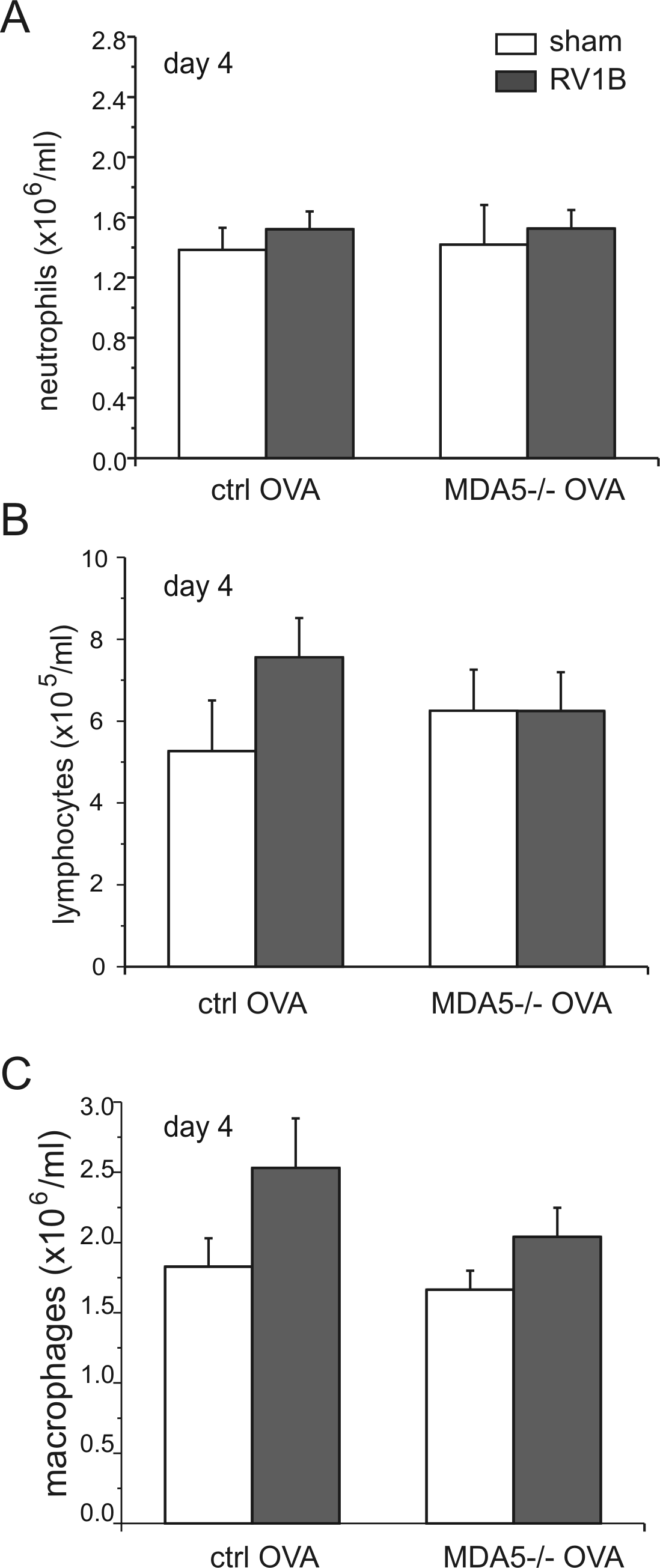

Supplement: Figure S5 — Late lung neutrophil, lymphocyte and macrophage counts in OVA-treated RV1B-infected MDA5−/− mice: histology and airways responsiveness. MDA5−/− mice and their control mice were sensitized and challenged with OVA and then infected with RV1B. Ninety-six h after infection, lungs were digested by collagenase. A–C. The numbers of infiltrated neutrophils, lymphocytes, and macrophages were counted. Data represent mean±SEM for 4–7 mice. (TIF) [file ppat.1002070.s005.tif]

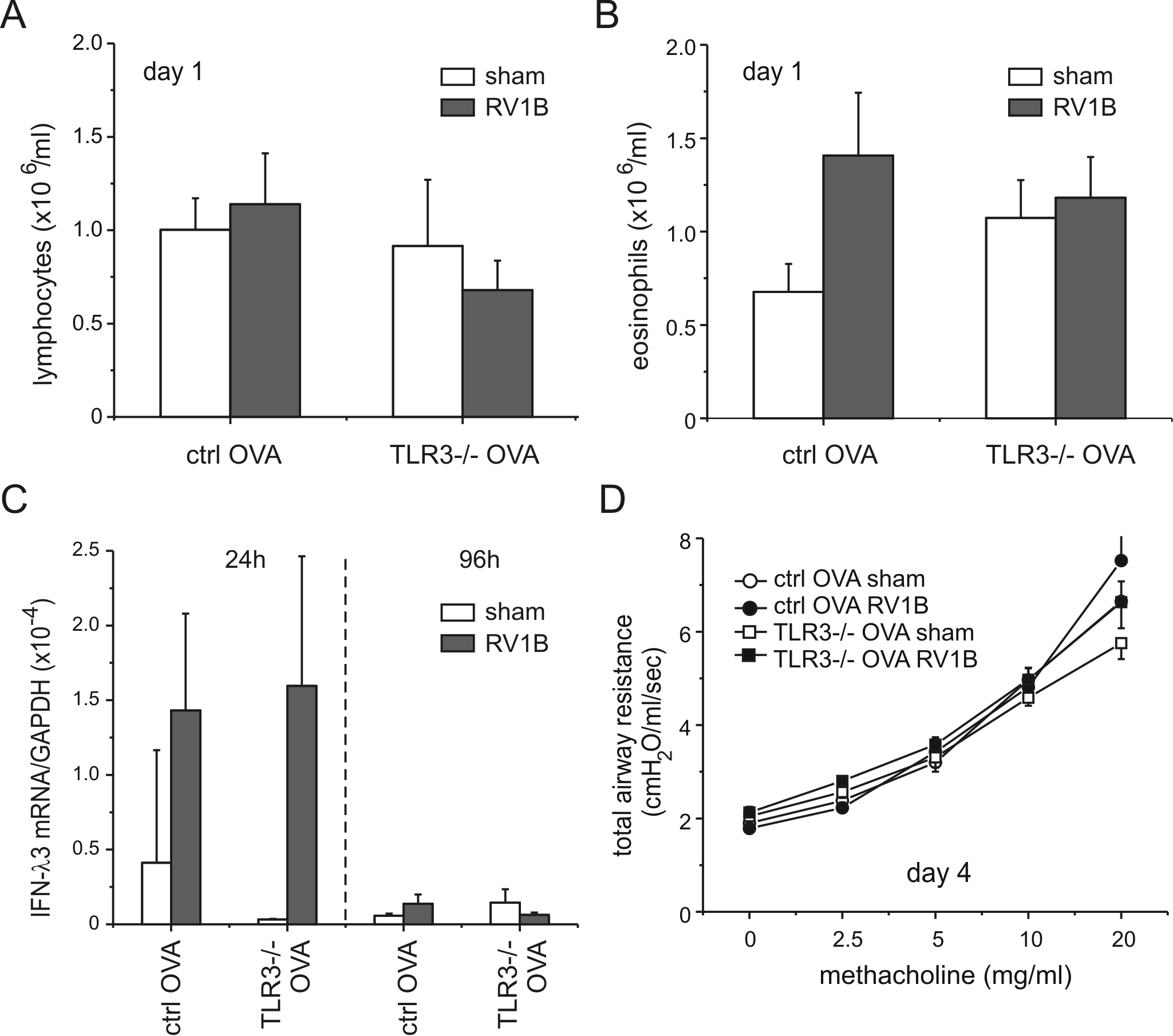

Supplement: Figure S6 — Cell counts, IFN-l3 mRNA levels and late airways responsiveness in OVA-treated RV1B-infected TLR3−/− mice. TLR3−/− mice and their control mice were sensitized and challenged with OVA and then infected with RV1B. A, B. Twenty-four h after infection, lungs were digested by collagenase and the numbers of infiltrated lymphocytes and eosinophils counted. C. The mRNA expression of IFN-λ3 was determined by qPCR. Expression was normalized to GAPDH. D. Airway cholinergic responsiveness 96 h after RV1B infection. Data represent mean±SEM for 4 mice. (TIF) [file ppat.1002070.s006.tif]
